# Autophagy regulates MK-2206-induced LDL receptor expression and cholesterol efflux pathways

**DOI:** 10.1371/journal.pone.0338076

**Published:** 2025-12-04

**Authors:** Hilde Sundvold, Thea Bismo Strøm

**Affiliations:** Unit for Cardiac and Cardiovascular Genetics, Department of Medical Genetics, Oslo University Hospital, Oslo, Norway; Nathan S Kline Institute, UNITED STATES OF AMERICA

## Abstract

**Background:**

Hypercholesterolemia remains a key risk factor for atherosclerotic cardiovascular disease (CVD). The clearance of low-density lipoprotein (LDL) particles from plasma, primarily mediated by LDL receptor (LDLR) activity, is an established target of lipid-lowering therapies. Enhancing reverse cholesterol transport via high-density lipoprotein and modulating cholesterol efflux from macrophages further complements atherogenic risk reduction. Enhancing LDLR expression and supporting effective cholesterol efflux via ATP binding cassette subfamily A member 1 (ABCA1) are therefore essential therapeutic targets for CVD prevention. Recent studies implicate autophagy in lipid and cholesterol metabolism. This study examines the influence of autophagy on LDLR and ABCA1 expression in hepatocytes after treatment with the AKT inhibitors MK-2206 and triciribine.

**Methods:**

Autophagy was disrupted pharmacologically using SBI-0206965 and genetically via short-interfering RNA (siRNA) targeting autophagy-related genes *ATG5* and *ATG7* in HepG2. Stable knockout (KO) HAP1 cell lines for *ATG5* and *ATG7* were generated by CRISPR to ensure complete abrogation of autophagy. The possible effect of SREBP2 silencing on MK-2206-induced LDLR expression was assessed in HepG2 cells. Quantitative analyses included measurement of ABCA1, LDLR and MAP1LC3B (LC3B) expression at protein and mRNA levels, in addition to *ULK1* and *SQSTM1* (*p62*) mRNA levels.

**Results:**

MK-2206 administration increased hepatic LDLR and the autophagy marker LC3B. Triciribine did not show evidence of autophagy induction, and neither AKT inhibitors modified ABCA1 expression. Inhibition of autophagy, either by SBI-0206965 or by siRNA targeting ATG5 and ATG7, reduced the MK-2206-mediated LDLR upregulation by approximately 50% in HepG2. In KO-ATG5/ATG7 HAP1 cells, the MK-2206-induced LDLR expression decreased by 70% compared to wild-type cells, and ABCA1 expression was abolished.

**Conclusion:**

Both pharmacological and genetic impairment of autophagy attenuate the LDLR-inducing effects of MK-2206, supporting a role for autophagy in the regulation of cholesterol metabolism. The substantial reduction of ABCA1 expression in autophagy-deficient cells further indicates that autophagy is involved in cholesterol efflux regulation.

## Introduction

Autophagy is an evolutionarily conserved catabolic process responsible for maintaining cellular homeostasis by eliminating or recycling dysfunctional organelles and proteins via cytosolic sequestration followed by lysosomal degradation [1,2]. Basal autophagy contributes to lipid metabolism, including processes collectively termed lipophagy, and has identified its involvement in various aspects of lipid metabolism—such as lipogenesis, lipolysis, fatty acid oxidation, ketogenesis, and cholesterol efflux [3–5]. Noteworthy, loss of autophagy in adipose tissue leads to increased adipose mass, impaired adipogenesis, and elevated inflammation, implying autophagy as necessary for normal adipocyte differentiation and lipid turnover [6]. Further, hepatic autophagy deficiency impairs triglyceride mobilization and bile acid homeostasis, and may result in hepatic steatosis and insulin resistance [7–9]. Finally, autophagy is responsive to hepatic cholesterol levels and while sterol depletion initiates autophagy, an accumulation of hepatic cholesterol inhibits the pathway [9,10]. These studies indicate that the regulatory scope of autophagy in lipid metabolism is broader than previously thought and remains incompletely defined.

The low-density lipoprotein receptor (LDLR) is central to the cellular uptake of plasma LDL cholesterol, with elevated levels of the latter associated with increased cardiovascular disease (CVD) risk. Upon cellular sterol depletion, sterol regulatory element-binding protein 2 (SREBP2) is routed to the Golgi apparatus and cleaved to generate its mature form. Mature SREBP2 translocate to the nucleus, activating genes containing sterol-regulatory elements (SREs) required for fatty acid and cholesterol synthesis, as well as cholesterol uptake via LDLR induction [10–12]. MK-2206, an allosteric AKT inhibitor with oral bioavailability, has been evaluated for anticancer properties in preclinical and clinical contexts [13–15] and has also demonstrated a capacity to increase LDLR expression and function [16]. The induction of LDLR by MK-2206 requires SREBP2 but is not dependent on intracellular cholesterol status, distinguishing it from statins [16]. Triciribine, an alternative AKT inhibitor, similarly promotes LDLR expression and enhances LDL uptake. Notably, this effect stems from increased LDLR mRNA stability rather than transcriptional activation via SREBP2 [17,18].

The PI3K/AKT/mTOR signaling cascade regulates multiple cellular processes, including transcription, translation, metabolism, proliferation, cell cycle progression, and apoptosis [19–21]. This pathway is a principal target for therapeutic intervention and serves as a key negative regulator of autophagy [22]. Lipids and their metabolic enzymes can influence autophagy by modulating PI3K/AKT/mTOR activity [9,22] and evidence indicates that AKT and mTOR inhibition prompts autophagy initiation [23]. Studies further show that cholesterol-lowering agents reduce AKT-mTOR signaling while increasing autophagy in hepatocytes [9,24–26]. Inhibition of AKT, either using MK-2206 or RNA interference, was initially found to induce autophagy in human glioma cells, and subsequent reports describe similar effects in various other cell types, including hepatocarcinoma cells [13–15]. While studies suggest triciribine suppresses AKT signaling and induces autophagy in cardiomyocytes and T-cell acute lymphoblastic leukemia cell lines, its role in hepatic autophagy remains to be elucidated [27,28].

Cholesterol transport by high-density lipoprotein (HDL) particles to the liver for excretion serves as the primary mechanism for removing excess cholesterol from non-hepatic tissues, including atherogenic macrophages. Deficiency in ATP-binding cassette subfamily A1 (ABCA1), a key protein mediating intracellular cholesterol efflux to HDL, leads to arterial cholesterol deposition and is associated with early-onset CVD [29]. In macrophages, autophagy can be activated by atherogenic lipids such as oxidized LDL (oxLDL) and 7-ketocholesterol, one of the primary oxysterols in oxLDL [30]. Enhanced autophagy activation is also necessary for delivering lipid droplets to lysosomes for hydrolysis to generate free cholesterol for ABCA1-dependent cholesterol efflux in macrophage foam cells, thus reducing macrophage foam cell formation and production of atherosclerotic lesions [5].

Autophagy involves more than 30 conserved autophagy-related proteins (ATGs), from yeast to mammals. Two ubiquitin-like conjugation systems—the ATG12-ATG5-ATG16L1 complex and LC3B-I/LC3B-II formation—are required for phagophore elongation and autophagosome–lysosome fusion [31,32]. Conventional autophagy relevant to hepatic lipid metabolism depends on ATG5 and ATG7, while additional proteins including ULK1, beclin-1, LC3B, and SQSTM1/p62 (p62) are important for autophagy initiation and nucleation [3,33–36]. The mTORC1 complex negatively regulates autophagy, partly by phosphorylating ULK1 [37,38].

Given observations of increased SREBP2 proteolytic processing following MK-2206 treatment—independent of SRE induction—alongside concurrent autophagy activation, the objective of this study was to assess whether autophagy modulates LDLR expression directly. The study also examined if triciribine influences autophagy in hepatocytes and the association with LDLR induction. Additionally, modulation of ABCA1-expression was evaluated in autophagy-modulated cells subjected to AKT inactivation.

## Materials and methods

### Cell culture

HepG2 cells (European Collection of Cell Cultures, Salisbury, UK) were maintained on collagen-coated vessels (BD Biosciences, San Jose, CA) in HyClone Minimum Essential Medium (MEM; GE Healthcare Life Sciences, Pittsburgh, PA) supplemented with 10% fetal bovine serum (FBS), 2 mM L-glutamine (Sigma-Aldrich, St. Louis, MA), 50 U/mL penicillin, 50 µg/mL streptomycin (GE Healthcare Life Sciences), and non-essential amino acids (Biowest, Nuaillé, France). HAP1 cells (a kind gift from Dr. Noam Zelcer, University of Amsterdam) were cultured in Gibco IMDM with GlutaMAX Supplement (Thermo Fisher Scientific, Waltham, MA) with 10% fetal bovine serum, 50 U/mL penicillin, 50 µg/mL streptomycin, and non-essential amino acids. All cells were incubated at 37°C in a humidified atmosphere with 5% CO₂.

### Drug treatments

All drugs were prepared in DMSO, with a final concentration of DMSO at 0.1% (v/v) for all conditions, including controls. MK-2206–2HCl (MK-2206; S1078) and triciribine (TCN; S1117) from Selleck Chemicals (Houston, TZ), and Rapamycin (R8781) and SBI-0206965 (SBI; SML1540) from Sigma-Aldrich were utilized as indicated in the figure legends. The final concentration of each component was carefully optimized to retain cell viability over 70% (Fig S1A, S2, S4A, S5 in S1 Supporting Information).

### Gene knockdown

For siRNA-mediated knockdown, 0.3x10^6^ HepG2 cells were seeded per well in 6-well plates (area 9.5 cm^2^) overnight in complete MEM medium. Cells were transfected the following day with 40 pmol gene-specific siRNAs per well targeting human *SREBF2* (*SREBP2*; SI00065856), *ATG5* (SI02655310), and *ATG7* (SI02655373) from Qiagen (Hilden, Germany) for 14–16 hours using 3 µL Lipofectamine RNAiMAX (Invitrogen, Carlsbad, CA). Non-targeting AllStars negative control siRNA (SI04380467) was used as negative control (mock). The *SREBP2* siRNA SI00065856 was selected from a FlexiTube GeneSolution package of four preselected siRNAs (Qiagen) according to knock-down efficiency in HepG2 (data not shown). The validation of siRNA *ATG5* and *ATG7* has previously been described [39]. Knock-down efficiency was confirmed for each biological replicate (four in total) with qPCR probes and by Western blot analyses using immunospesific antibodies.

### MTT cell viability assay

Cell viability was assessed using an MTT assay (ab211091; Abcam, Cambridge, UK) according to the manufacturer’s guidelines with modifications. Briefly, 0.06x10^6^ HepG2 cells were seeded out in 24-well plates (area 1.9 cm^2^) instead of 96-wells plates in complete MEM medium and treated with the indicated agents or vehicle for 14–16 hours. The following day, medium was replaced with serum-free medium, and MTT reagent was added for 3 h at 37°C. After incubation, MTT solvent was added and cells incubated for an additional 15 min. Absorbance was measured at 590 nm using Synergy H1 microplate reader (BioTek, Winooski, VT) to determine cell viability relative to the vehicle sample.

### CRISPR/Cas9-mediated gene editing

HAP1 cells are widely used in functional genetic studies due to their rapid proliferation and haploid genome, which allows for efficient phenotype expression following single-allele modification [40]. CRISPR/Cas9 editing of HAP1 cells was performed as described previously [41]. Briefly, 0.8x10^6^ HAP1 cells were seeded out in 60 mm culture dishes (area 21 cm^2^) in complete IMDM medium and incubated at 37°C in a humidified atmosphere with 5% CO₂ overnight. The following day, medium was changed to IMDM without penicillin and streptomycin and the cells were co-transfected with pCMV-Cas9-GFP and pU6-gRNA (Sigma-Aldrich) at a 1:1 ratio (4.5 µg DNA in total) using Lipofectamine™ 2000 Transfection Reagent (Invitrogen) at a ratio of 1 µL transfection reagent to 0.3 µg DNA. The transfection medium was changed to complete IMDM medium after 6h and cells were harvested 48 h post-transfection. GFP-positive single-cell clones were sorted by a BD FACS Melody cell sorter (Becton and Dickinson, San Jose, US) at the Oslo University Hospital Flow Cytometry Core Facility and expanded. Verified HAP1 *ATG5* knockout lines were used as templates for *ATG5/ATG7* double knockout generation (KO-ATG5/ATG7). Gene editing was confirmed by Sanger sequencing of the targeted exons and protein expression analyses of ATG5 and ATG7 compared to non-transfected cells by Western blot. gRNA target sequences and induced frameshift are listed in Table S1 in S1 Supporting Information.

### Western blot analysis and antibodies

Cells were frozen to −80°C for at least 30 minutes prior to lysis at 4°C for 20 minutes in Triton X-100 buffer (20 mM Tris pH 7.5, 100 mM NaCl, 1% Triton X-100, 10 mM EDTA) containing protease inhibitors. Lysed cells were centrifugated at 15 000 RPM for 15 minutes at 4°C to remove cell debris. Protein concentrations were determined via Pierce™ BCA Protein Assay (Thermo Fisher Scientific). Equal protein amounts were separated on 4–20% TGX-SDS-PAGE gels at 200 V for 30 minutes then transferred to PVDF membranes by semidry blotting utilizing the Trans-Blot Turbo Transfer System from Bio-Rad (Hercules, CA). PVDF-membranes were blocked in 5% non-fat dry milk prior to incubation with the target-specific antibody at 4°C overnight. Antibodies used included rabbit mAb ATG7 (D12B11) (8558; 1:500) and rabbit mAb ATG5 (2630; 1:1000) from Cell Signaling (Danvers, MA), mouse mAb SREBP2 (557037; 1:250) from BD Pharmingen (San Diego, CA), rabbit mAb LC3B (ab48394; 1:500), rabbit mAb LDL Receptor (ab52818; 1:5000, HepG2) and mouse mAb ABCA1 (ab18180; 1:5000) from Abcam, and rabbit mAb LDL Receptor (20R-LR002; 1:500, HAP1) from Biosynth (Berkshire, UK). Cell type-specific anti-LDLR antibodies were used after validation, based on differential epitope recognition in HepG2 and HAP1 cells. β-Actin served as a loading control and was detected using rabbit mAb β-Actin (ab213262; 1:7500) from Abcam. Primary antibody detection was performed using HRP-conjugated secondary antibodies—anti-rabbit IgG (#7074S) or anti-mouse IgG (#7076S) from Cell Signaling. The secondary antibodies were incubated at 4 °C overnight, and hybridization signals were visualized using the SuperSignal™ West Dura Extended Duration Substrate (Thermo Fisher Scientific). The band intensities were quantified by the use of Chemidoc Touch Imaging System (Bio-Rad).

### Quantitative real-time PCR

Drug treated HepG2 cells, originally seeded out at 0.4x10^6^ in 12-well plates (area 3.5 cm^2^), were frozen to −80°C for at least 30 minutes prior to RNA-isolation with the QIAamp RNA Isolation Kit (Qiagen). Low protein contamination was validated upon measuring A260/A280: ~ 2.0 using a NanoDrop spectrophotometer (Thermo Fisher Scientific). 300 ng total RNA was reverse transcribed using the AffinityScript QPCR cDNA Synthesis Kit (Agilent Technologies, Santa Clara, CA). cDNA was diluted 1:5 with RNAse free water and 9 ng served as template for quantitative real-time polymerase chain reaction (qPCR) using Brilliant III Ultra-Fast QPCR Master Mix on Mx3005P QPCR system (Agilent technologies) using the following PrimeTime Predesigned human qPCR Assays (Integrated DNA Technologies, Coralville, IA): *LDLR* (Hs.PT.58.14599757), *GAPDH* (Hs.PT.39a.22214836), *ATG5* (Hs.PT.58.2898629), *ATG7* (Hs.PT.58.20398086), *SREBF2* (Hs.PT.58.45335433), *MAP1LC3B* (*LC3B*) (Hs.PT.5827295455.g), *ULK1* (Hs.PT.58.26275611), *ABCA1* (Hs.PT.58.27452429) and *p62* (Hs.PT.58.39829257). Reactions were run in technical duplicates or triplicates and included internal cDNA controls in each run. Target mRNA levels were normalized to GAPDH, and relative quantification was calculated using the 2^ − ΔΔCt method [42].

### Statistical analysis

Data are presented as mean with standard deviation (SD), unless otherwise specified. *P* values were calculated using Microsoft Excel with a two-sample t-test assuming equal or unequal variance, depending on results from an F-test checking for equal variances. A significance level of *P* < 0.05 was used. All experimental replicates refer to biological replicates (n = 3–4) as specified in the figure legends.

## Results

### MK-2206 induces autophagy and LDLR Expression in HepG2 cells

**Fig 1:**
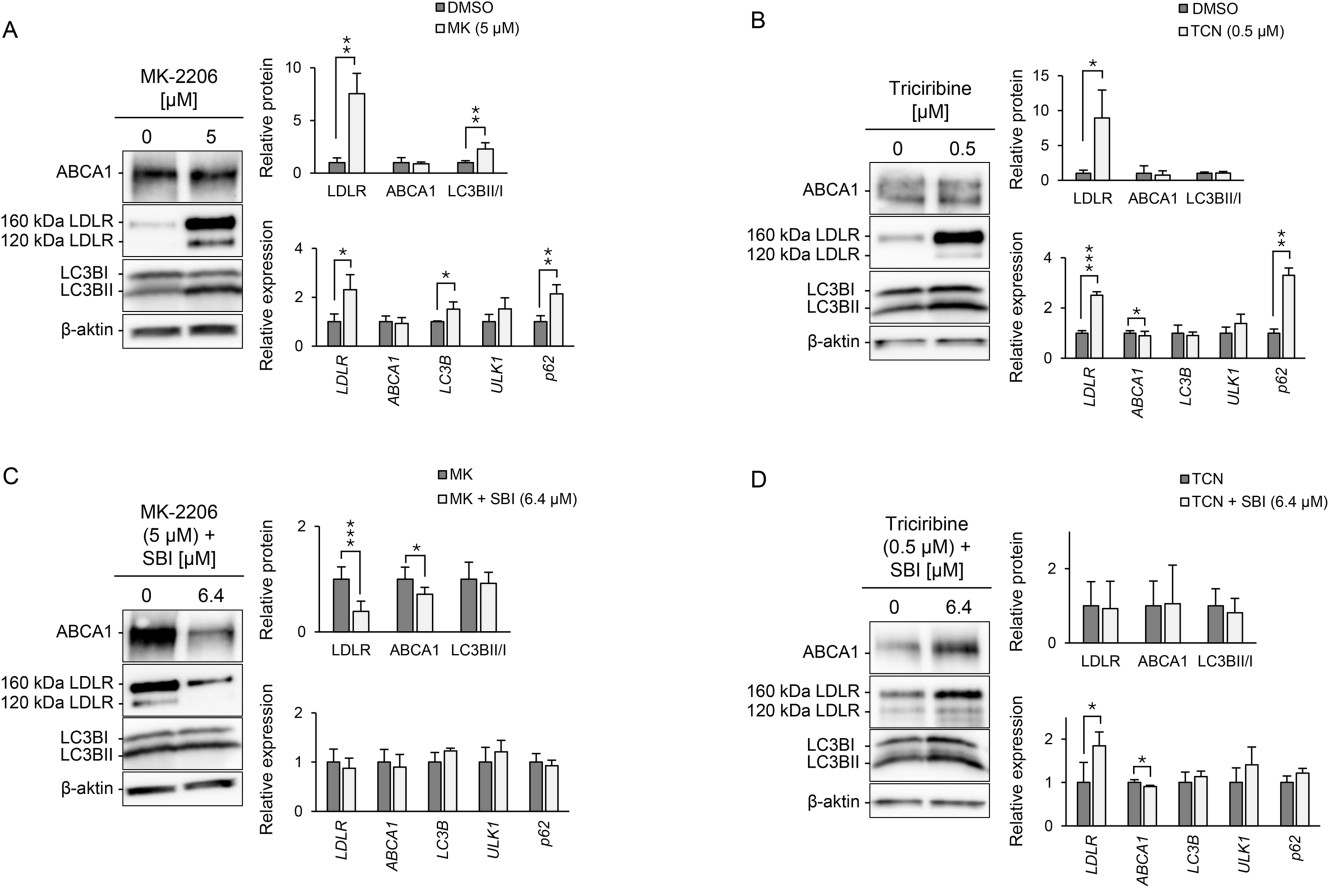
The effect of MK-2206 on LDLR in HepG2 cells is modulated by autophagy. The autophagy-inducing effects of MK-2206 (MK) and triciribine (TCN), along with their impact on LDLR and ABCA1 expression, were evaluated in HepG2 cells. HepG2 cells were cultured for 14-16 h in the absence (DMSO) or the presence of **A)** MK-2206 (5 µM) or B) triciribine (0.5 µM) alone, or C-D) pretreated with SBI-0206965 (SBI; 6.4 µM) for 30 minutes prior to administration. Protein levels of ABCA1, LDLR, LC3BI and LC3BII were analyzed by immunoblotting, showing one representative western blot. Quantified values were normalized to β-actin and expressed relative to levels observed in untreated cells (n = 4). Quantitative PCR was used to assess gene expression of *ABCA1*, *LDLR*, *LC3B*, *ULK1* and *p62*, with values normalized to *GAPDH* and expressed relative to levels observed in untreated cells (n = 3). Error bars represent SD. *P < 0.05, **P < 0.01, and ***P < 0.001 compared with untreated cells.

MTT assays were performed upon component treatment to assess optimal concentrations and monitor cell viability (Fig S1 in S1 Supporting Information). Neither agents appreciably affected cell viability under the experimental conditions used, consistent with that previously reported [16,17]. The effects of MK-2206 and triciribine on the expression of LDLR, ABCA1, LC3B-II/I and their mRNA, along with *p62* and *ULK1* were assessed in HepG2 cells (Fig 1A, Fig S2 in S1 Supporting Information). Both compounds significantly upregulated LDLR expression; however, neither agent altered ABCA1 levels under these conditions. Notably, MK-2206 treatment resulted in a dose-dependent increase in *LC3B*, *ULK1*, and *p62* mRNA, suggesting enhanced autophagosome formation, initiation of autophagy, and increased autophagic demand, respectively (Fig 1A, Fig S2A in S1 Supporting Information). Conversely, triciribine selectively elevated *p62* expression without affecting LC3B-II/I or *ULK1* levels, indicating its action may involve stress response pathways distinct from canonical autophagy (Fig 1B, Fig S2B in S1 Supporting Information).

To further characterize the specificity of autophagy-related effects, rapamycin—a well-established mTOR pathway inhibitor—was employed as a control. Rapamycin treatment elevated LC3B-II/I and *ULK1* expression, alongside modest increases in LDLR and ABCA1 protein (Fig S3 in S1 Supporting Information). Unlike MK-2206 and triciribine, rapamycin did not alter *p62* levels, which may indicate maintenance of autophagic flux.

### SBI-0206965 modulates the effect of MK-2206 on LDLR

To evaluate autophagy dependency, the impact of the ULK1 inhibitor SBI-0206965 on LDLR regulation was investigated through co-administration with either MK-2206 or triciribine (Fig 1C, 1D). SBI-0206965 alone showed no effect on LDLR, ABCA1, or LC3B-II/I (Fig S4A). At higher concentrations, SBI-0206965 modestly increased *ULK1* mRNA and reduced *ABCA1* and *LDLR*, without changes in *LC3B*, or *p62* mRNA. Co-administration of MK-2206 and SBI-0206965 attenuated the LDLR response by 60% but retained the effect on LC3B-II/I expression (Fig 1C). In contrast, triciribine enhanced LDLR expression independently of SBI-0206965 co-administration (Fig 1D), further supporting the autophagy-independent mechanism of triciribine. Both MK-2206 and triciribine retained their induction of *p62* mRNA upon SBI-0206965 administration. Additionally, co-treatment of SBI-0206965 with rapamycin attenuated the LDLR-inducing response of rapamycin, while further increasing the expression of *LC3B* and *ULK1*, indicating an accumulation of these markers (Fig S4B in S1 Supporting Information)

### ATG5/ATG7 are essential for autophagy and ABCA1 expression

**Fig 2:**
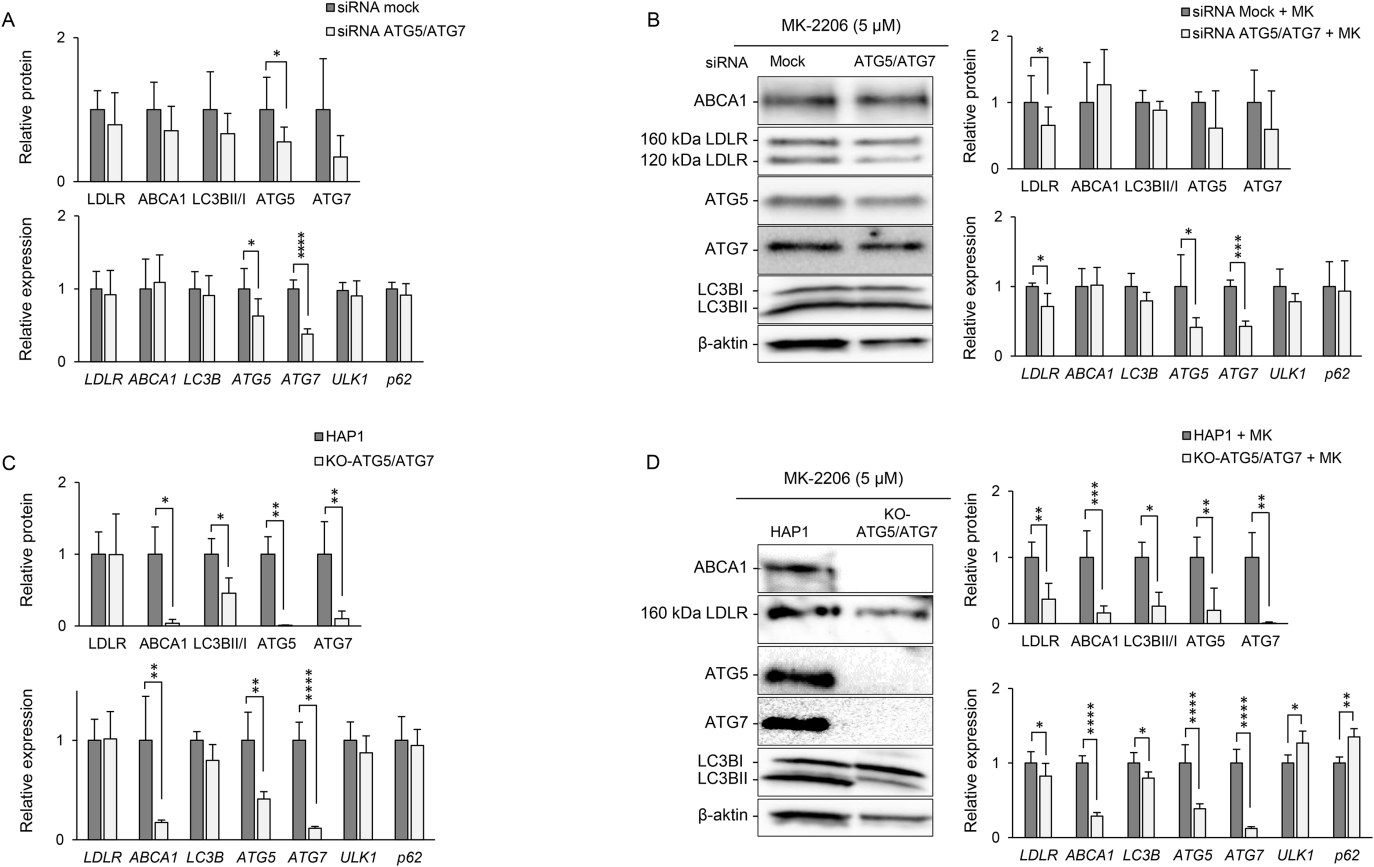
ATG5 and ATG7 are essential for autophagy and ABCA1 expression. The effect of genetic inhibition of conventional autophagy by knockdown of ATG5 and ATG7 was evaluated transient in HepG2 and by CRISPR-targeting in HAP1 cells. **A)** HepG2 cells were targeted with siRNA against control (mock) or ATG5 and ATG7 for 24 h alone or B) prior to MK-2206 treatment for 14-16 **h. C)** HAP1 wild-type cells (HAP1) or CRISPR-targeted ATG5 and ATG7 (KO-ATG5/ATG7) were cultured untreated or D) in the presence MK-2206 (5 µM) for 14-16 **h.** Protein levels of ABCA1, LDLR, LC3BI, LC3BII, ATG5 and ATG7 were analyzed by immunoblotting, showing one representative western blot. Quantified values were normalized to β-actin and expressed relative to levels observed in untargeted cells (n = 4). Quantitative PCR was used to assess gene expression of *ABCA1*, *LDLR*, *LC3B*, *ATG5, ATG7, ULK1* and *p62*, with values normalized to *GAPDH* and expressed relative to levels observed in untargeted cells (n = 3). Error bars represent SD. *P < 0.05, **P < 0.01, ***P < 0.001 and ****P < 0.0001 compared to untargeted cells.

While ABCA1 protein was induced by rapamycin but not by MK-2206 or triciribine (Fig S2, Fig S3 in S1 Supporting Information), combined MK-2206/SBI-0206965 treatment resulted in a 25% reduction in ABCA1 protein (Fig 1C). To clarify potential off-target effects or incomplete inhibition, subsequent experiments focused on evaluating the roles of ATG5 and ATG7 in MK-2206-mediated modulation and ABCA1 expression. HepG2 cells transiently depleted for ATG5 and ATG7 (20–70% knockdown) showed no significant effect on ABCA1, LDLR, LC3B-II/I, *ULK1*, or *p62* expression (Fig 2A). The MK-2206-induced increase in LDLR was reduced in ATG5/ATG7-depleted HepG2 cells, consistent with SBI-0206965 co-treatment (Fig 2B).

To circumvent incomplete knockdown, CRISPR-generated KO-ATG5/ATG7 HAP1 cells were analyzed. In untargeted HAP1 cells, MK-2206 increased LDLR and LC3B-II/I protein, and *ABCA1* and *ULK1* mRNA, with no significant effect on ABCA1 protein (Fig S5A in S1 Supporting Information). ATG7 protein increased after MK-2206, while its mRNA was unchanged. In HAP1, MK-2206 did not affect *p62* expression, indicating that LDLR induction in HepG2 cells is independent of the marked increase observed in *p62*. KO-ATG5/ATG7 HAP1 cells displayed ~90% reduced ATG5/ATG7 protein and 50–90% lower mRNA compared with wildtype (Fig 2C, Fig S5B in S1 Supporting Information). Expression of *LDLR*, *ULK1*, and *p62* mRNA was similar in untargeted and KO-ATG5/ATG7 HAP1 cells (Fig 2C). LC3B-II conversion was significantly impaired, and *LC3B* mRNA was reduced, consistent with defective autophagy. ABCA1 protein was nearly undetectable in KO cells, according to a 90% reduction in *ABCA1* mRNA (Fig 2C, Fig S5B in S1 Supporting Information). Despite some residual effect, MK-2206-mediated induction of LDLR was significantly reduced in KO-ATG5/ATG7 HAP1 cells (Fig 2D). These cells also exhibited a modest increase in *p62* following MK-2206 treatment, compared with untargeted HAP1 (Fig 2D).

### LDLR induction by MK-2206 is partially dependent on SREBP2

**Fig 3:**
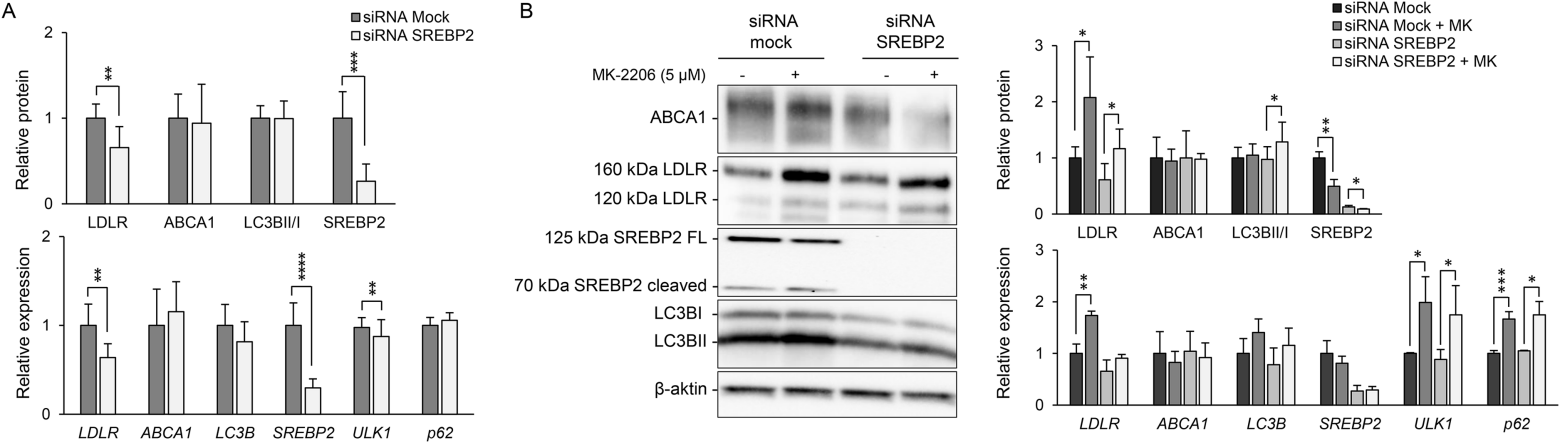
LDLR is induced by MK-2206 in a SREBP2-dependent manner. The effect of SREBP2 depletion on MK-2206 (MK) induction of LDLR and autophagy was evaluated transient in HepG2: **A)** HepG2 cells were targeted with siRNA against control (mock) or SREBP2 for 24 h alone or B) prior to MK-2206 (5 µM) treatment for 14-16 **h.** Protein levels of ABCA1, LDLR, LC3BI, LC3BII and SREBP2 (full length (FL) and cleaved form) were analyzed by immunoblotting, showing one representative western blot. The full length 125 kDa form of SREBP2 was used for quantification due to antibody sensitivity. Quantified values were normalized to β-actin and expressed relative to levels observed in untargeted cells (n = 4). Quantitative PCR was used to assess gene expression of *ABCA1*, *LDLR*, *LC3B*, *SREBP2, ULK1* and *p62*, with values normalized to *GAPDH* and expressed relative to levels observed in untargeted cells (n = 3). Error bars represent SD. *P < 0.05, **P < 0.01, ***P < 0.001 and ****P < 0.0001 compared to untargeted cells.

Finally, since MK-2206-induced LDLR expression has been shown to depend on SREBP2, we aimed to assess how SREBP2 depletion affects both LDLR levels and autophagy markers simultaneously. HepG2 cells were transiently depleted for SREBP2 with 70% efficiency (Fig 3A). Both LDLR protein and mRNA levels decreased by around 35%. Interestingly, MK-2206 retained its LDLR-inducing effect at the protein level in SREBP2-depleted cells, although *LDLR* mRNA was abolished (Fig 3B). MK-2206 increased expression of *ULK1* and *p62* equally in both untargeted, and SREBP2-depleted cells, indicating retained induction of autophagy.

## Discussion

**Fig 4:**
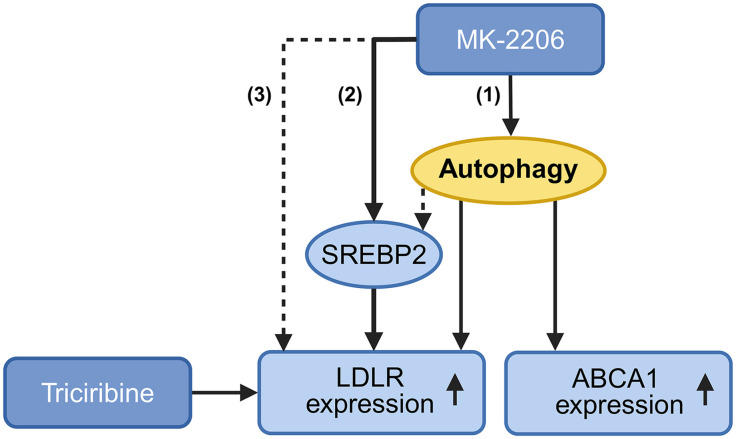
Schematic representation of how autophagy regulates cholesterol metabolism in hepatocytes.

Hyperlipidemia significantly increases cardiovascular risk, and a growing body of evidence linking autophagy to cholesterol metabolism highlights its therapeutic potential in hyperlipidemia. Increased expression of LDLR – the key protein in LDL clearance- is correlated with increased autophagy when the AKT-mTOR pathway is inactivated, supporting the role of autophagy in hepatic LDLR regulation [43]. In this study, we have evaluated the effects of the AKT inhibitors MK-2206 and triciribine, with a particular focus on how their modulation of LDLR and ABCA1 relates to autophagy. Whereas MK-2206 increased both autophagy markers and LDLR expression, triciribine elevated LDLR levels without influencing autophagy (Fig 4).

Schematic representation of how autophagy is suggested to regulate cholesterol metabolism in hepatocytes. The AKT inhibitor MK-2206 promotes LDLR expression via multiple pathways: (1) by inducing autophagy, possibly mediated via SREBP2; (2) by directly upregulating SREBP2 independently of autophagy; and (3) independently of both autophagy and SREBP2. In contrast, triciribine also increases LDLR expression but does so independently of both autophagy and SREBP2, indicating a distinct mechanism of action. Additionally, ABCA1 expression is completely abolished in autophagy-deficient cells, indicating a critical role for autophagy in the regulation of cholesterol efflux. Created in https://BioRender.com.

Supporting these observations, rapamycin—a potent autophagy inducer—elicited a similar response to MK-2206 in terms of LDLR and LC3B expression, although the increase was more modest. Notably, MK-2206’s effect on LDLR was reduced by 70% in cells with impaired autophagy, suggesting the involvement of multiple regulatory pathways. While previous studies have established a link between LDLR and autophagy, particularly through the PI3K/AKT/mTOR signaling pathway, this study reveals a previously underappreciated role of autophagy as an upstream regulator of key cholesterol-handling proteins in hepatocytes. Unlike prior research that primarily focused on how LDLR activity or cholesterol levels influence autophagy, this work demonstrates that autophagy is essential for the induction of LDLR expression in response to AKT inhibition. Importantly, we demonstrate that LDLR upregulation by MK-2206 is partially dependent on functional autophagy, whereas triciribine induces LDLR independently of autophagic flux. This differential regulation has not been previously described and highlights distinct mechanistic pathways for LDLR modulation.

Furthermore, our findings reveal that ABCA1 expression is critically dependent on intact autophagy machinery, suggesting a novel role for autophagy in cholesterol efflux regulation in hepatocytes. By using both pharmacological and genetic approaches, including CRISPR-mediated knockout of ATG5 and ATG7, this study provides robust evidence that autophagy is a critical determinant of cholesterol homeostasis, offering new insights with potential therapeutic relevance for cardiovascular disease.

Autophagy is a complex, tightly regulated process involving multiple checkpoints. The conversion of LC3B-I to LC3B-II is a widely used marker of autophagy induction, though it does not discern between enhanced formation and reduced degradation of autophagosomes. Thus, additional markers are necessary to accurately monitor autophagic flux. ULK1 is critical for autophagy initiation, while p62 serves as a cargo adaptor directing ubiquitinated proteins to autophagosomes. Normally, p62 is degraded alongside its cargo during autophagy; when autophagy is inhibited, p62 accumulates, indicating impaired flux. In our study, both MK-2206 and triciribine induced *p62* mRNA expression in HepG2 cells, suggesting an association with oxidative stress, given p62’s known role in the Keap1-NRF2 pathway, which governs cytoprotective responses to oxidative and electrophilic stress [44]. Moreover, p62 is itself a target of NRF2, creating a feedback mechanism that may amplify cytoprotective responses. Notably, the increase in *p62* mRNA observed with MK-2206 treatment was evident in HepG2 but not in HAP1 cells, pointing to a cell-type-specific effect. MK-2206 treatment in hepatocytes increased levels of LC3B-II/I, *ULK1*, and *p62*, suggesting an induction of early-stage autophagy with a possible blockage at later stages. This is supported by the stable *ULK1* mRNA levels and the accumulation of *p62*. Nonetheless, in HAP1 cells, MK-2206 enhanced LDLR expression independently of *p62* induction, indicating that the LDLR response does not depend on the oxidative stress pathway mediated by p62.

Although triciribine significantly upregulated LDLR protein and mRNA in HepG2 cells, consistent with previous reports [17], neither LC3B-II/I protein nor *LC3B* mRNA levels were altered. Triciribine’s effect on LDLR was also independent of chemical impaired autophagy by SBI-0206965, supporting the existence of an alternative, autophagy-independent mechanism. Previous studies have noted that triciribine-induced LDLR upregulation is limited to hepatocytes, suggesting tissue-specific regulation [17]. Reports of triciribine-induced autophagy in other cell types, such as cardiomyocytes and leukemia cell lines, but not in liver, further support the possibility of cell-type-dependent effects [27,28]. Thus, autophagic flux was indicated by increased LC3B-II/I and *ULK1* levels and only MK-2206 and rapamycin, but not triciribine, altered these. Triciribine’s effect on LDLR, previously observed only in hepatocytes and to rely on an increased LDLR mRNA stability thus seems independent of both autophagy and SREBP2.

Cholesterol homeostasis is tightly regulated by intracellular cholesterol levels, with decreased cholesterol activating autophagy through PIP3-dependent mechanisms and SREBP2. MK-2206 enhances the proteolytic processing of SREBP2, a key transcription factor in cholesterol metabolism, but this response is reportedly cholesterol-independent [16]. Consequently, prior studies indicate that MK-2206 upregulates LDLR via SREBP2-dependent mechanisms. Consistent with these findings, our data show that SREBP2 knockdown abrogates the LDLR response to MK-2206 at the mRNA level. However, the LDLR protein response to MK-2206 persists. This may reflect incomplete SREBP2 depletion or a longer half-life of *SREBP2* mRNA but could also arise from a SREBP2-independent, autophagy-related, mechanism of action. Full clarification may require complete SREBP2 knockout studies, preferably in hepatocytes.

In macrophages, autophagy is required for trafficking lipid droplets to lysosomes, facilitating cholesterol efflux via ABCA1 and limiting foam cell formation—a critical step in atherogenesis [5,45]. The role of autophagy in cholesterol efflux has been reported to be cell type specific and regulated by both the level of autophagy and the mechanism that triggers autophagy [45]. Notably, AKT- and mTOR1c-inhibition has been shown to increase cholesterol efflux in HepG2 cells, and MK-2206 has been reported to induce cholesterol efflux in vascular smooth muscle cells [46,47]. Herein, ABCA1-expression remained stable upon MK-2206 administration to HepG2 cells, but was however roughly 25% reduced upon impaired autophagy. Although MK-2206 increased *ABCA1* mRNA but not its protein in HAP1 cells, the ABCA1 protein was almost undetectable in autophagy-impaired cells compared to untargeted cells, indicating that autophagy is essential for ABCA1 expression and function. Oxidative stress responses are known to suppress LXR/RXR signaling, which is a key transcriptional activator of ABCA1 expression [48]. Disrupted canonical autophagy in KO-ATG5/ATG7 HAP1 cells may lead to accumulation of damaged organelles and misfolded proteins, which would activate oxidative stress responses. Moreover, an alternative ATG5-ATG7 independent autophagy pathway which bypasses LC3B lipidation has been postulated [36,49]. This pathway may not support lipid droplet clearance or cholesterol trafficking effectively, potentially leading to feedback suppression of ABCA1 due to intracellular cholesterol accumulation. Although MK-2206 did not affect *p62* expression, indicative of a potential oxidative stress response, the reduced ABCA1 expression in autophagy deficient KO-ATG5/ATG7 HAP1 cells strongly support a role for ABCA1 in autophagy-dependent cholesterol efflux.

Emerging evidence highlights the pivotal role of cellular stress responses, particularly endoplasmic reticulum (ER) stress and oxidative stress, in regulating autophagy and metabolism. As such, Sahoo et al. recently reported significant upregulation of ER stress-related genes such as *XBP1*, *HERPUD1*, and *SELENOS* in blood cells from septic neonatal foals, correlating with oxidative stress and disrupted cellular homeostasis [50]. These findings suggest that ER stress may converge on the PI3K/AKT/mTOR signaling axis, thereby modulating autophagic activity. Pathological conditions such as non-alcoholic fatty liver disease (NAFLD), atherosclerosis, and osteoporosis involve dysregulated lipid metabolism and oxidative stress, where the PI3K/AKT/mTOR signaling cascade plays a central role in modulating autophagy and cellular survival mechanisms, highlighting its pivotal role in cellular survival and disease progression [51–53]. Targeting autophagy for metabolic liver diseases is promising, as supported by preclinical models showing improvements in steatosis and injury with enhanced autophagy [54,55]. Our observations in hepatocytes reinforce the therapeutic relevance of targeting autophagy and oxidative stress pathways in lipid metabolic disorders. To date, several autophagy inducers and inhibitors have been identified and successfully implemented in disease models, including rapamycin, though most are in preclinical stages [56].

This study has several limitations. First, the study is restricted to in vitro models (HepG2 and HAP1 cell lines), and future in vivo validation is warranted to confirm the physiological relevance of the findings. Moreover, the analysis did not include upstream regulators of autophagy such as beclin-1, which could offer deeper insights into pathway activation. Imaging-based autophagy quantification visualizing autophagosome would further reinforce the results. Lastly, immunohistochemical staining for LDLR and ABCA1 in liver tissue would enhance the translational relevance of the results by linking cellular findings to tissue-level expression patterns.

Our findings underscore distinct mechanisms by which MK-2206 and triciribine modulate LDLR expression in hepatocytes. While MK-2206 acts through both autophagy-dependent and SREBP2-mediated pathways, triciribine induces LDLR independently of autophagic flux and SREBP2, previously demonstrated via enhanced LDLR mRNA stability. This divergence highlights the importance of considering compound-specific effects when targeting the AKT pathway for therapeutic modulation of cholesterol metabolism. Furthermore, although MK-2206 and triciribine both upregulate LDLR, only MK-2206 influences autophagy markers and ABCA1 expression, suggesting broader implications for cholesterol efflux and cellular stress responses. These differential effects emphasize the need for tailored approaches when evaluating AKT inhibitors in the context of lipid regulation and autophagy. In summary, these findings suggest that modulating autophagy could have physiological relevance towards increased understanding of factors and pathways causing hypercholesterolemia and may be a valuable strategy for future dyslipidemia treatment.

## Supporting information

S1 Supporting information Supplementary Table and figures. (PDF)

S2 Raw Images (PDF)
