# Supplementary material for: Autophagy regulates MK-2206-induced LDL receptor expression and cholesterol efflux pathways
Source: PLoS One. 2025 Dec 4;20(12):e0338076. doi: 10.1371/journal.pone.0338076 (PMC12677516; doi:10.1371/journal.pone.0338076)
Supplement: S1 Supporting information — (PDF) [file pone.0338076.s001.pdf]

# Supporting information

*Autophagy regulates MK-2206-induced LDL receptor expression and cholesterol efflux pathways*

Hilde Sundvold and Thea Bismo Strøm

**Table S1: gRNA used for CRISPR/Cas9-mediated gene editing**
Sequences of gRNA targeting ATG5 (NM_004849.1) and ATG7 (NM_006395.3) obtained from http://chopchop.cbu.uib.no/. Guide exon location, amino acid position, sequence and induced frameshift and are indicated.

| Name | Exon | Amino acids | Sequence | Induced frameshift |
|---|---|---|---|---|
| ATG5-gRNA | 6 | p.163-170 | ACCAGTTTTGGGCCATCAAT | c.206_221del |
| ATG7-gRNA | 13 | p.468-475 | TCAATAGGAAGACGACATCA | c.1402_1417del |

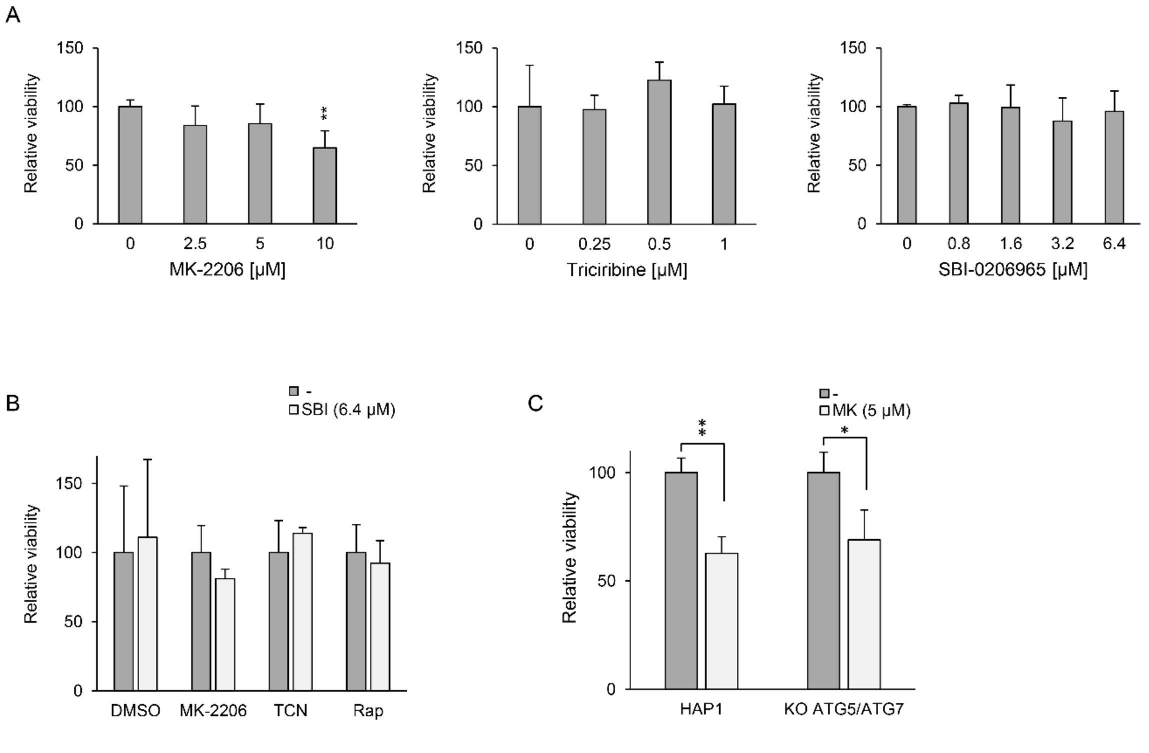


**Figure S1: Cell viability**
Cell viability was assessed using a MTT cell proliferation assay (ab211091; Abcam, Cambridge, UK) after component treatment; (A) Optimal working concentrations of MK-2206 (5 µM), triciribine (0.5 µM) and SBI-0206965 (6.4 µM) were selected based on viability of HepG2 cells cultured with increasing component concentrations for 14-16 h, (B) HepG2 cells cultured with-, or without, SBI-0206965 (6.4 µM) for 30 minutes and exposed to either vehicle (DMSO), MK-2206 (5 µM), triciribine (TCN; 0.5 µM) or rapamycin (Rap; 100 nM) for 14-16 h, (C) HAP1 wild-type cells or CRISPR-

targeted ATG5 and ATG7 (KO-ATG5/ATG7) cultured in the presence of DMSO or MK-2206 (5 µM) for 14-16 hours. Error bars represent SD. *$P$ < 0.05 and **$P$ < 0.01 compared with vehicle treated cells.

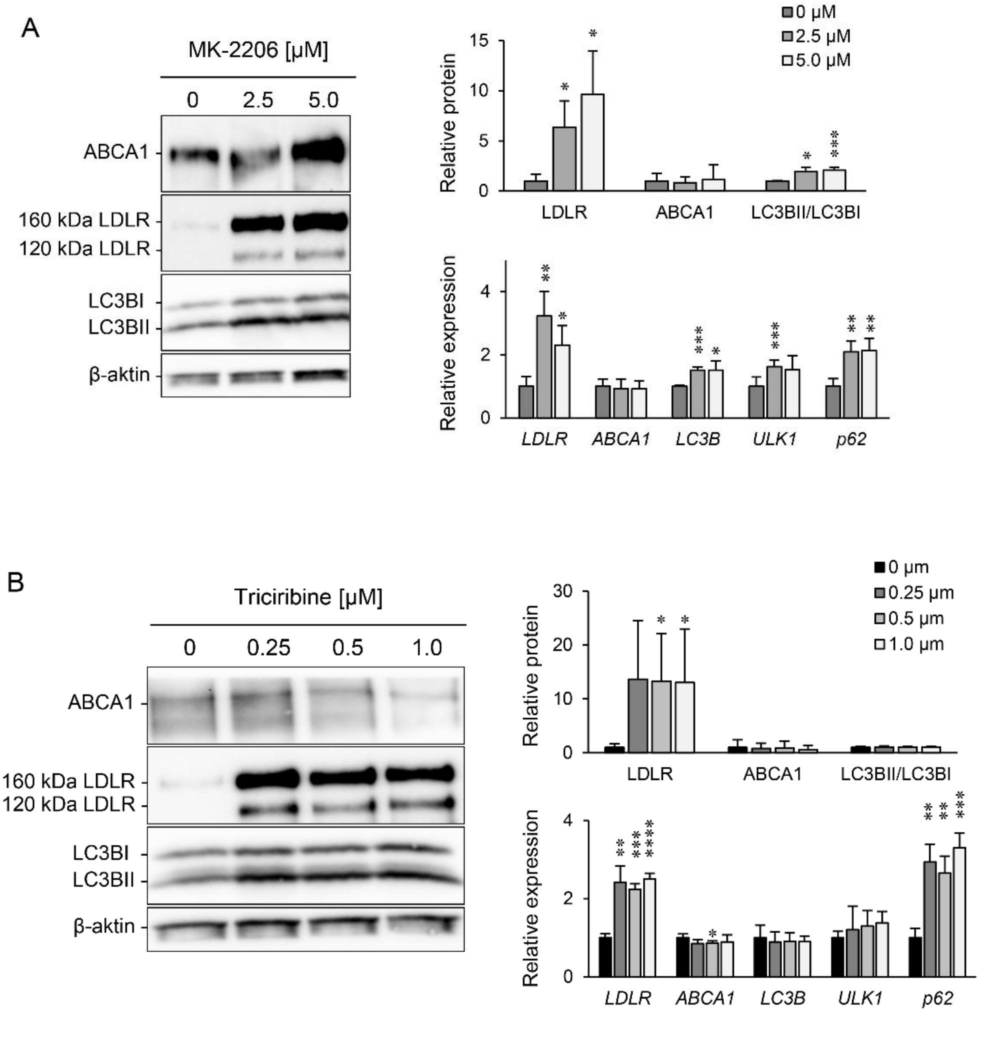


**Figure S2: Dose response effects of MK-2206 and triciribine in HepG2 cells**

HepG2 cells were cultured in the presence of AKT inhibitors A) MK-2206 or B) triciribine for 14-16 h at increasing concentrations. Protein levels of ABCA1, LDLR, LC3BI and LC3BII were analyzed by immunoblotting, showing one representative western blot. Quantified values were normalized to β-actin and expressed relative to levels observed in untreated cells. Quantitative PCR was used to assess gene expression of *ABCA1*, *LDLR*, *LC3B*, *ULK1* and *p62*, with values normalized to *GAPDH* and expressed relative to levels observed in untreated cells. Error bars represent SD. *$P$ < 0.05, **$P$ < 0.01, and ***$P$ < 0.001 compared with untreated cells.

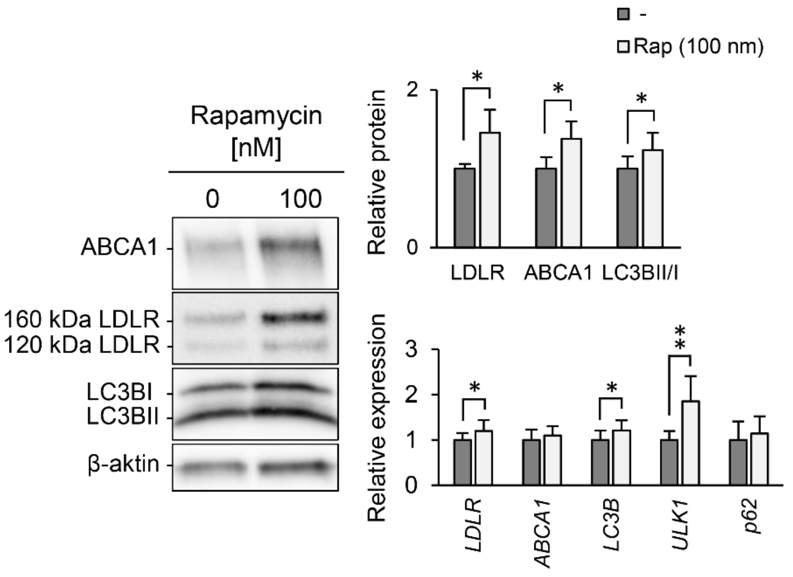


**Figure S3: Rapamycin induces autophagy and LDLR expression in HepG2 cells**
The impact of the mTOR pathway inhibitor rapamycin on autophagy and the expression of LDLR and ABCA1 was examined in HepG2 cells. HepG2 cells were cultured in the presence of vehicle (DMSO) or rapamycin (100 nM) for 14-16 h. Protein levels of ABCA1, LDLR, LC3BI and LC3BII were analyzed by immunoblotting, showing one representative western blot. Quantified values were normalized to β-actin and expressed relative to levels observed in untreated cells. Quantitative PCR was used to assess gene expression of *ABCA1*, *LDLR*, *LC3B*, *ULK1* and *p62*, with values normalized to *GAPDH* and expressed relative to levels observed in untreated cells. Error bars represent SD. **P* < 0.05, ***P* < 0.01, and ****P* < 0.001 compared with untreated cells.

A

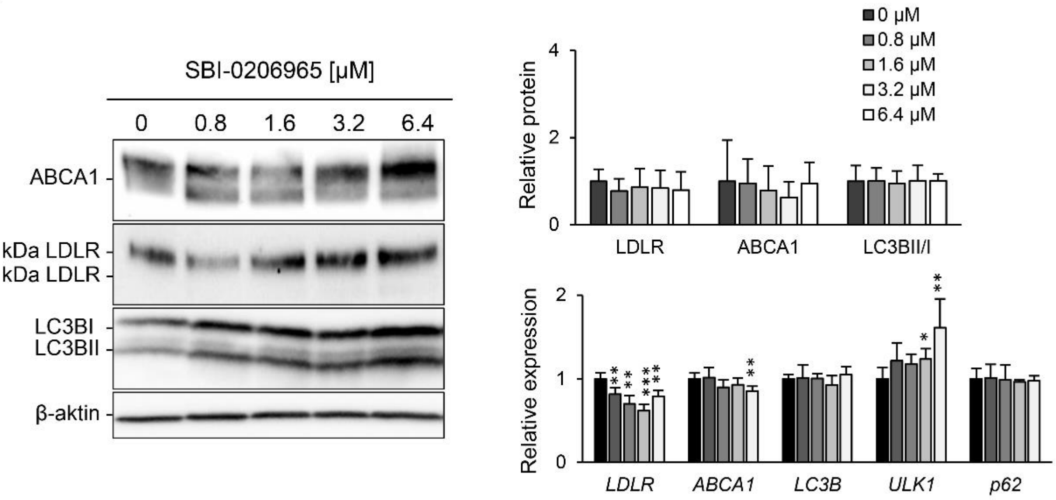

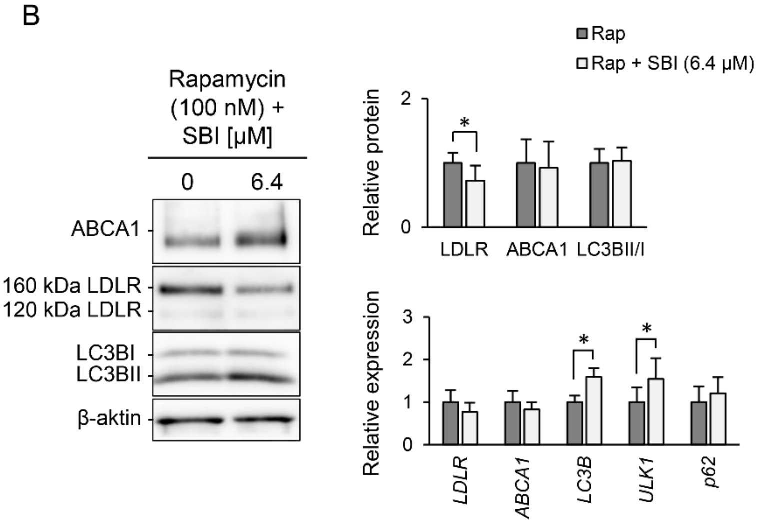


**Figure S4: Dose response effects of SBI-0206965 on expression in HepG2 cells**
Expression of LDLR and ABCA1 upon autophagy inhibition with SBI-0206965 was assessed in HepG2 cells: A) HepG2 cells were cultured in the presence of the indicated concentrations SBI-0206965 for 14-16 h, B) HepG2 cells were pretreated with or without SBI-0206965 (SBI; 6.4 µM) for 30 minutes prior to administration of rapamycin (100 nM). Protein levels of ABCA1, LDLR, LC3BI and LC3BII were analyzed by immunoblotting, showing one representative western blot. Quantified values were normalized to β-actin and expressed relative to levels observed in untreated cells. Quantitative PCR was used to assess gene expression of *ABCA1*, *LDLR*, *LC3B*, *ULK1* and *p62*, with values normalized to *GAPDH* and expressed relative to levels observed in untreated cells. Error bars represent SD. **P* < 0.05, ***P* < 0.01, and ****P* < 0.001 compared with untreated cells.

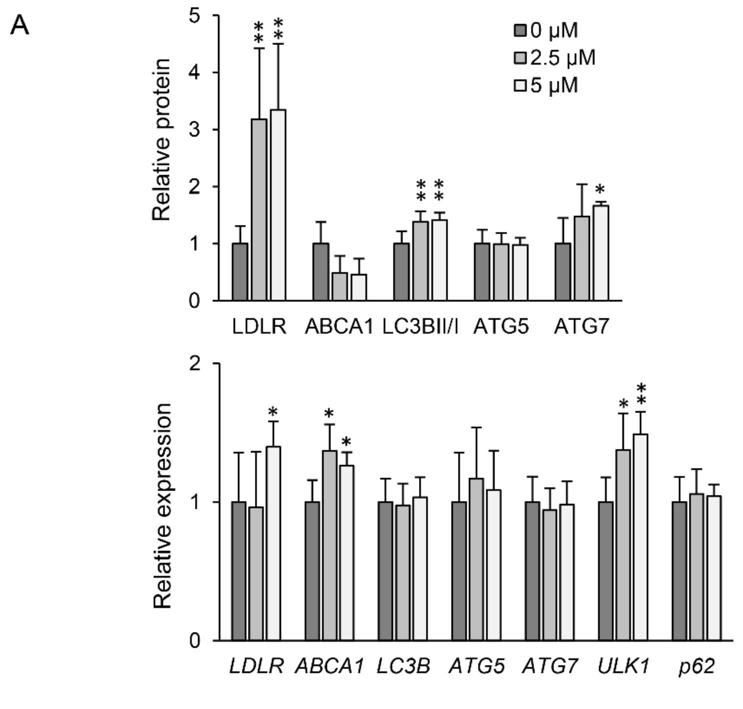

B

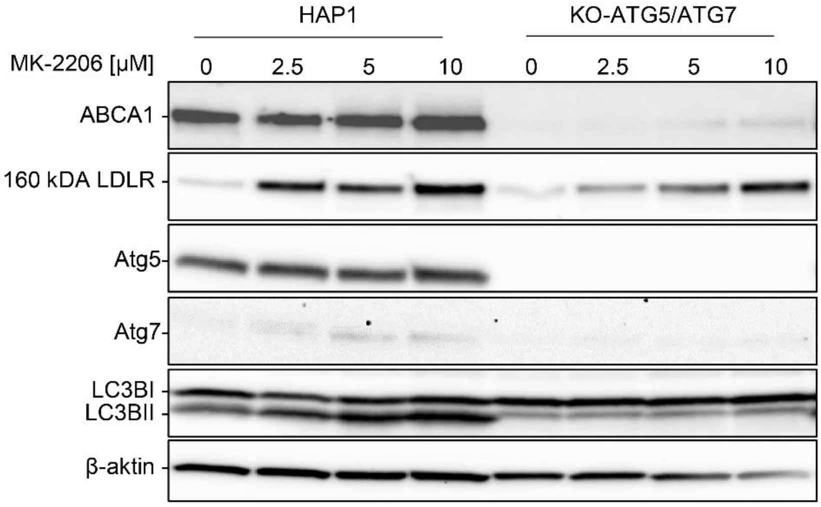


**Figure S5: Dose response effects of MK-2206 on expression in HAP1 cells**
The effect of MK-2206 was evaluated in HAP1 wild-type cells (HAP1) or CRISPR-targeted ATG5 and ATG7 (KO-ATG5/ATG7): A) Untargeted HAP1 cells were cultured in the presence of the indicated concentrations of MK-2206 for 14-16 hours. Protein levels of ABCA1, LDLR, LC3BI, LC3BII, ATG5 and ATG7 were analyzed by immunoblotting. Quantified values were normalized to β-actin and expressed relative to levels observed in untreated cells. Quantitative PCR was used to assess gene expression of *ABCA1*, *LDLR*, *LC3B*, *ATG5*, *ATG7*, *ULK1* and *p62*, with values normalized to *GAPDH* and expressed relative to levels observed in untreated cells. Error bars represent SD. $^{*}P < 0.05$ and $^{**}P < 0.01$ compared with untreated cells. B) One representative western blot of untargeted HAP1 or KO-ATG5/ATG7 cells cultured in the presence of the indicated concentrations of MK-2206, visualizing the prominent difference in protein expression of ABCA1, LC3BII, ATG5 and ATG7.
