# Supplementary material for: Autophagy regulates MK-2206-induced LDL receptor expression and cholesterol efflux pathways
Source: PLoS One. 2025 Dec 4;20(12):e0338076. doi: 10.1371/journal.pone.0338076 (PMC12677516; doi:10.1371/journal.pone.0338076)
Supplement: S2 Raw Images — (PDF) [file pone.0338076.s002.pdf]

Raw material

Autophagy regulates MK-2206-induced LDL receptor expression and cholesterol efflux pathways

Hilde Sundvold and Thea Bismo Strøm

Figure 1

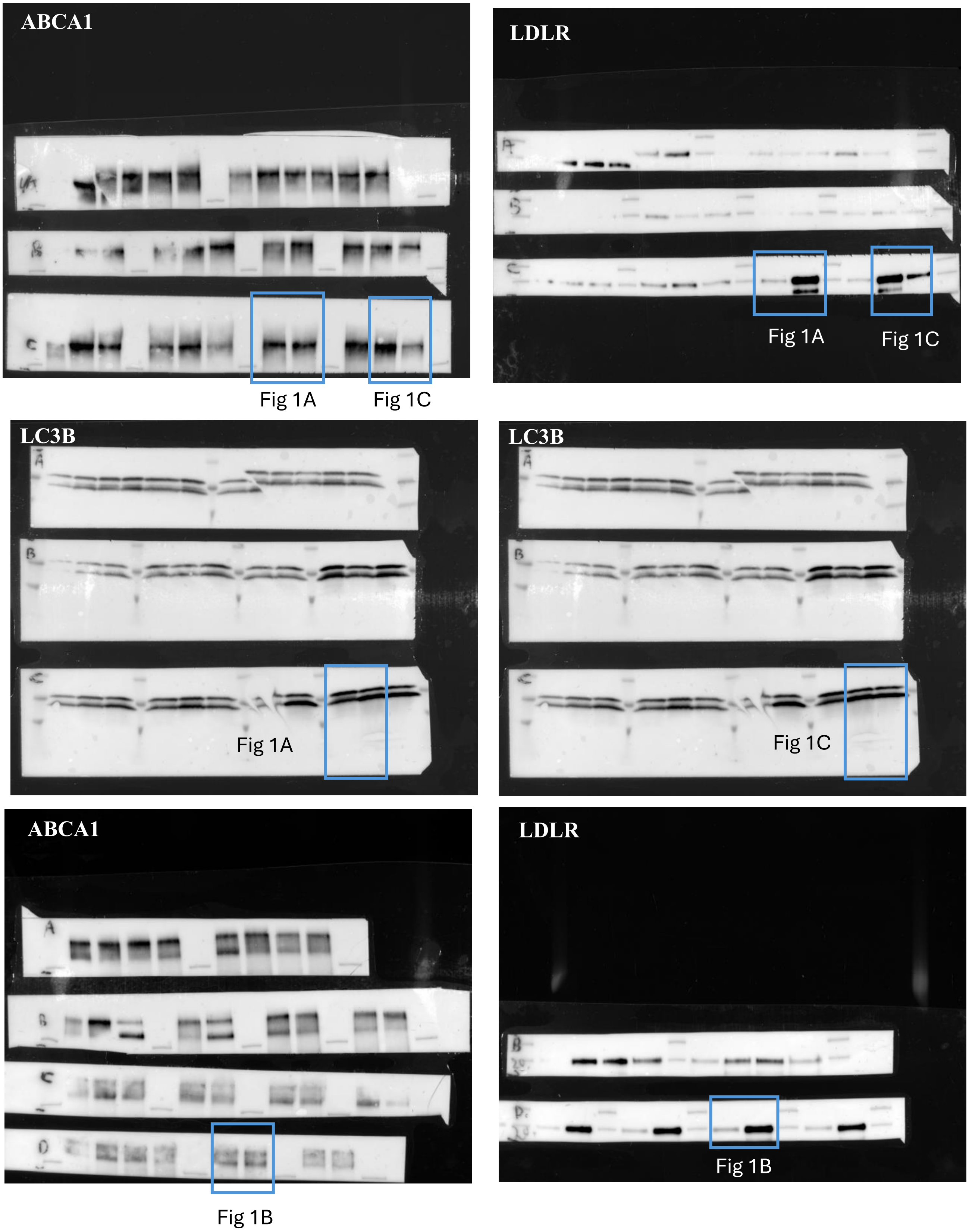

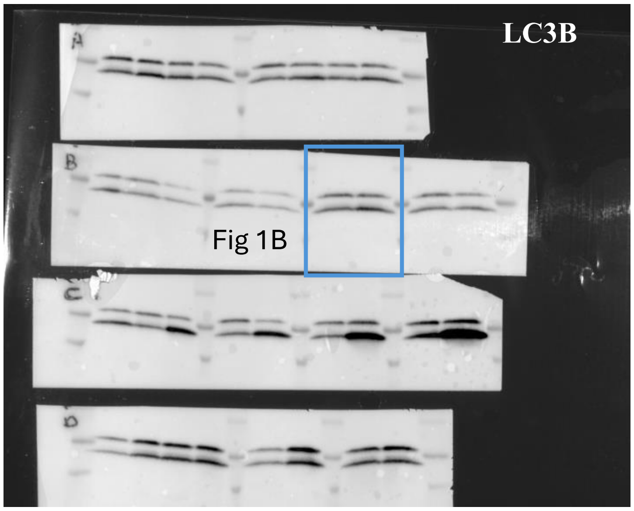

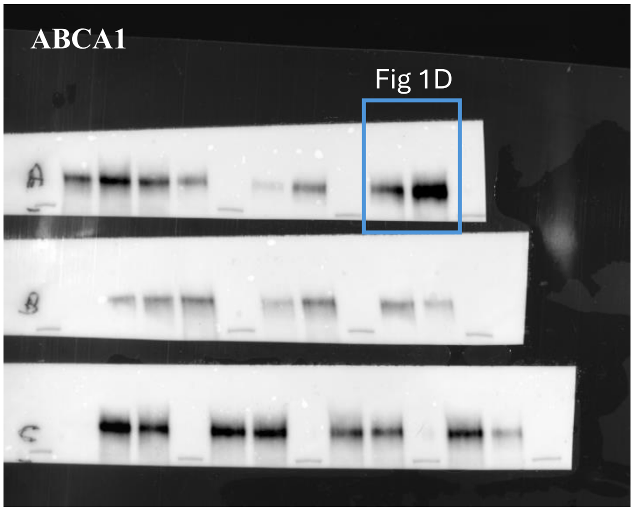

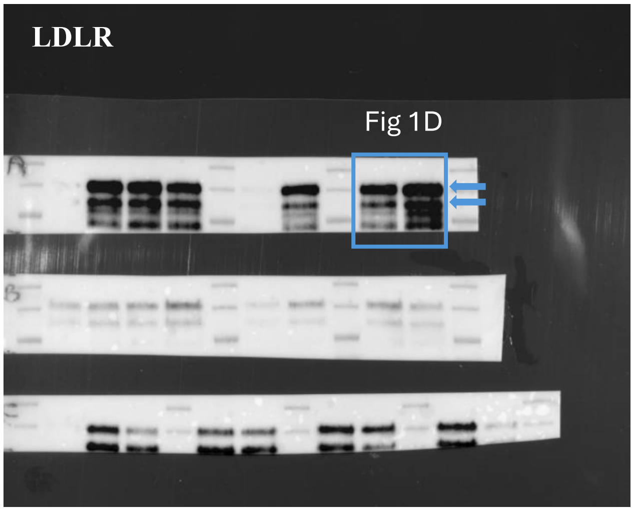

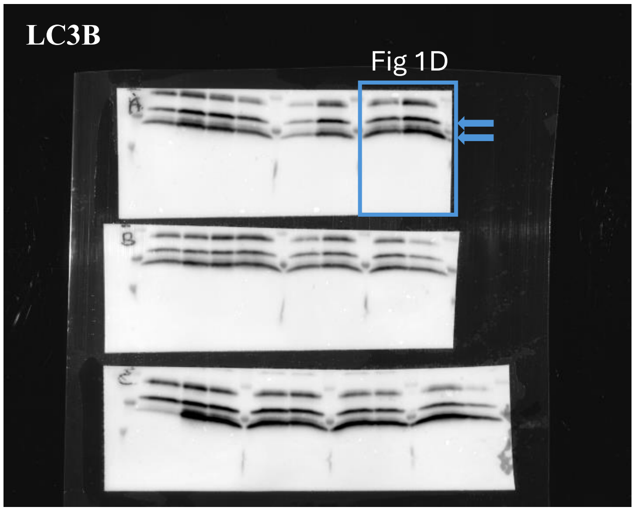

Figure 2

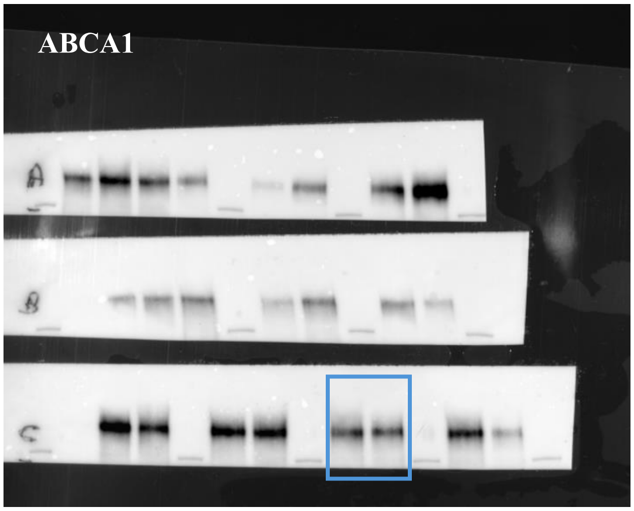

Fig 2A

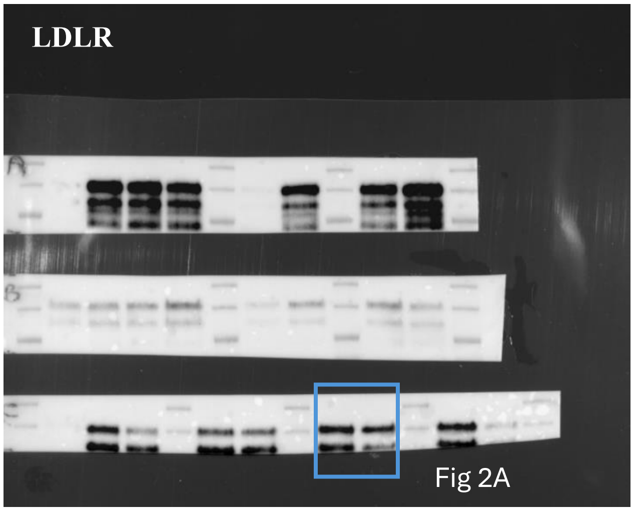

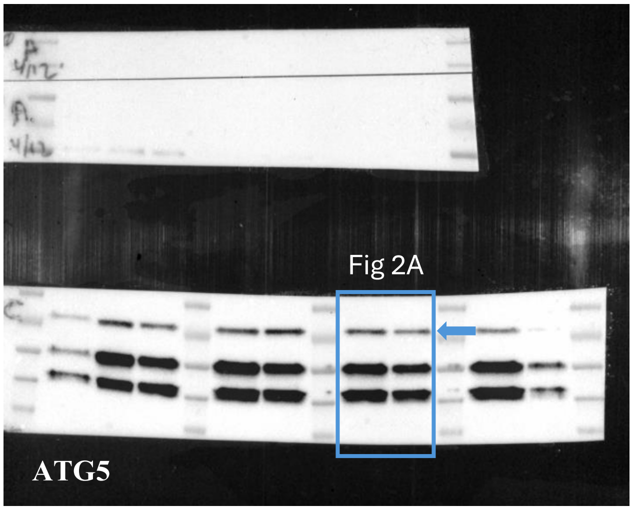
Fig 2A
ATG5

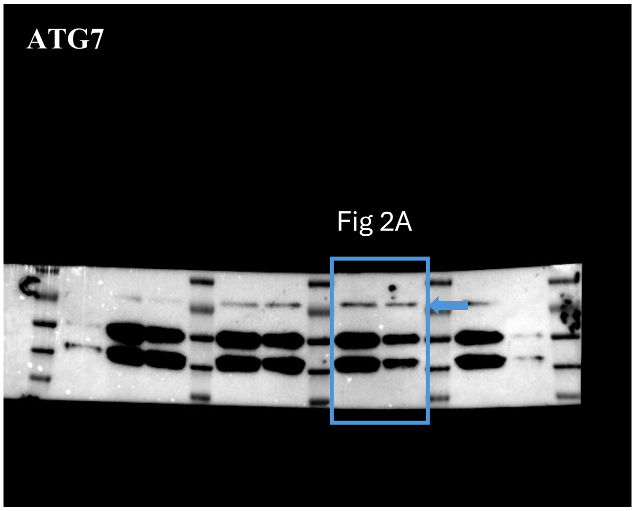
ATG7
Fig 2A

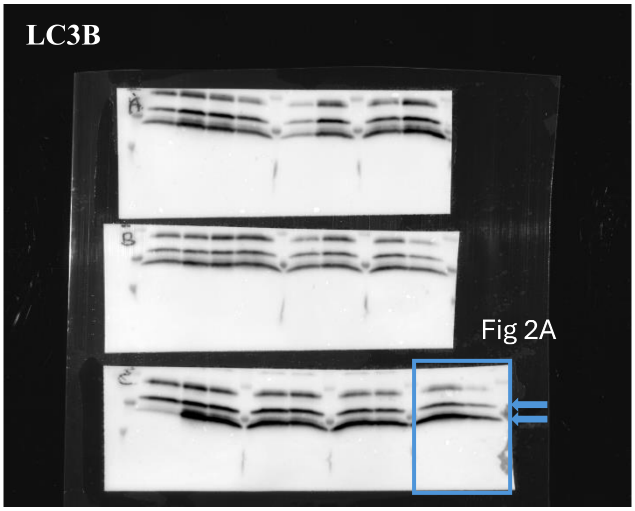
LC3B
Fig 2A

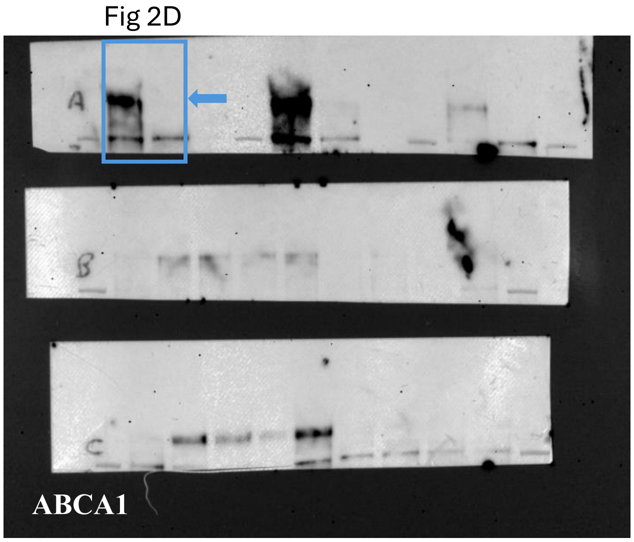
Fig 2D
ABCA1

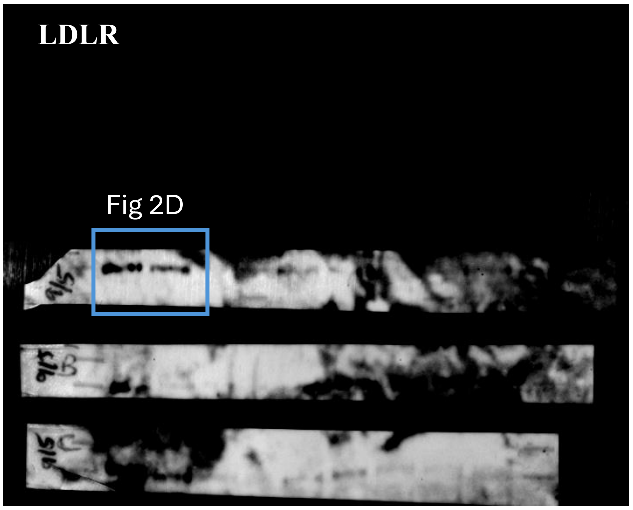
LDLR
Fig 2D

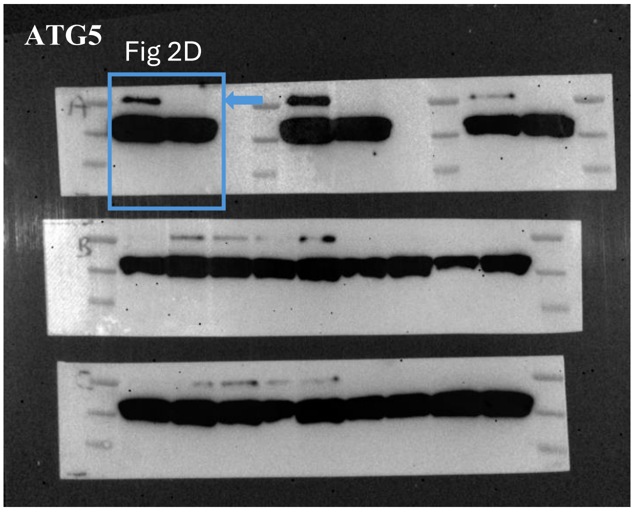
ATG5
Fig 2D

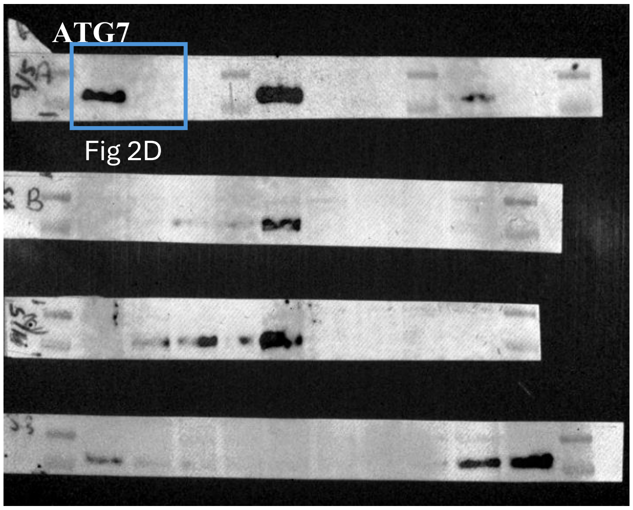
ATG7
Fig 2D

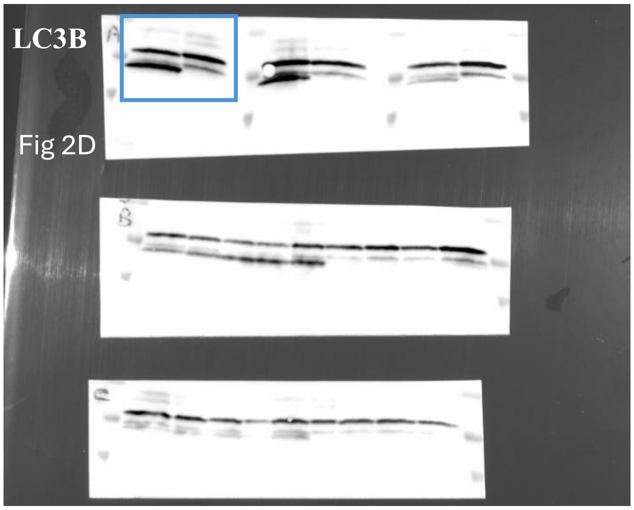
LC3B
Fig 2D

Figure 3

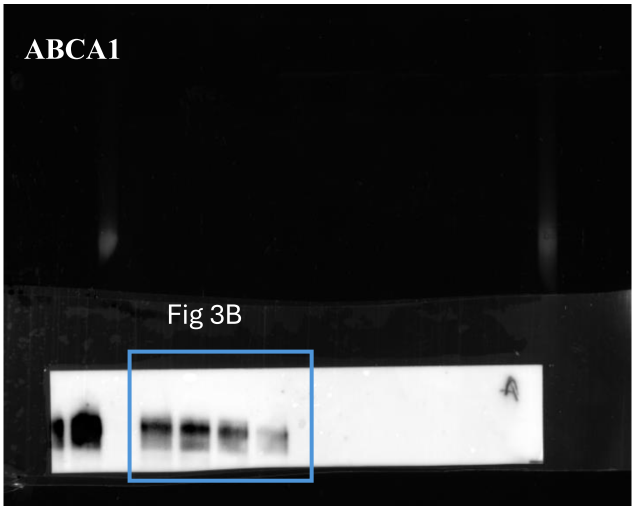
ABCA1
Fig 3B

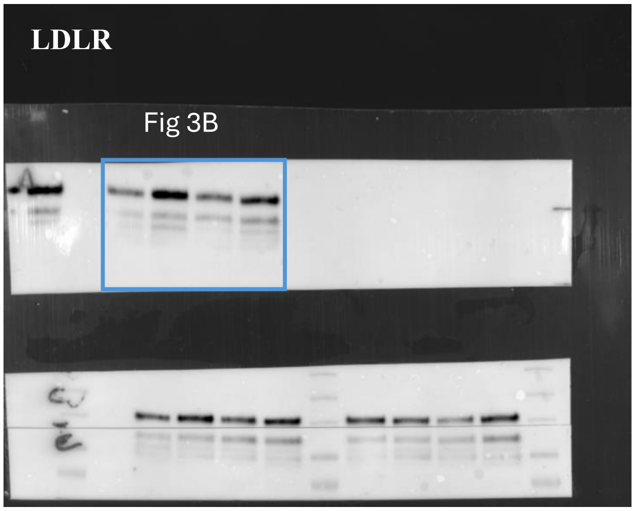
LDLR
Fig 3B

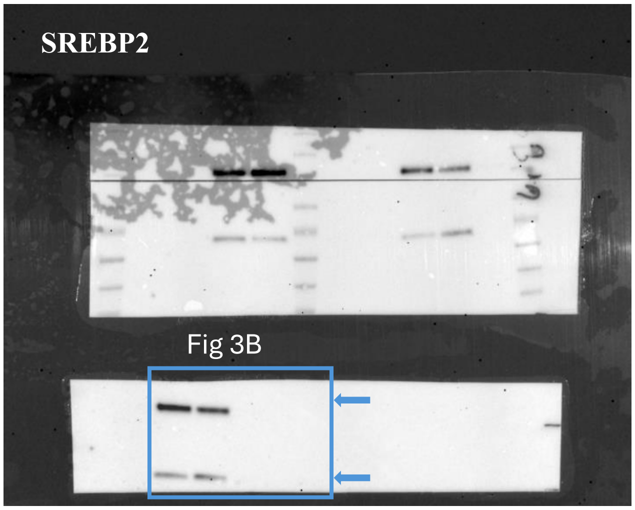
SREBP2
Fig 3B

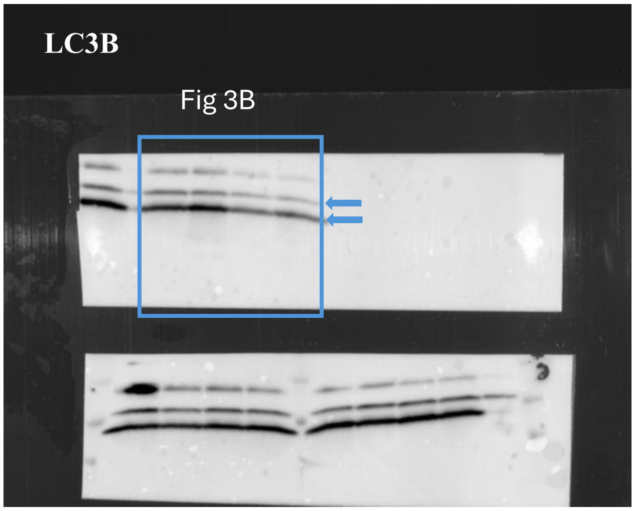
LC3B
Fig 3B
